# Influence of body composition assessment with bioelectrical impedance vector analysis in cancer patients undergoing surgery

**DOI:** 10.3389/fonc.2023.1132972

**Published:** 2023-09-05

**Authors:** Bin Cai, Lan Luo, Chenping Zhu, Liping Meng, Qing Shen, Yafei Fu, Mingjie Wang, Sue Chen

**Affiliations:** ^1^ Department of Quality Management, Sir Run Run Shaw Hospital, Zhejiang University School of Medicine, Hangzhou, Zhejiang, China; ^2^ Department of Clinical Nutrition, Shaoxing People’s Hospital, Shaoxing, Zhejiang, China; ^3^ School of Medicine, Shaoxing University, Shaoxing, Zhejiang, China

**Keywords:** body composition, bioelectrical impedance vector analysis, gastrointestinal cancer, nutritional status, malnutrition

## Abstract

**Background:**

Malnutrition is common in patients undergoing surgery for cancers and is a risk factor for postoperative outcomes. Body composition provides information for precise nutrition intervention in perioperative period for improving patients’ postoperative outcomes.

**Objection:**

The aim was to determine changes in parameters of body composition and nutritional status of cancer patients during perioperative period.

**Methods:**

A total of 92 patients diagnosed with cancer were divided into gastrointestinal and non-gastrointestinal cancer group according to different cancer types. The patients body composition assessed by bioelectrical impedance vector analysis (BIVA) on the day before surgery, postoperative day 1 and 1 day before discharge. The changes between two groups were compared and the correlation between body composition and preoperative serum nutritional indexes was analyzed.

**Results:**

The nutritional status of all patients become worse after surgery, and phase angle (PA) continued to decrease in the perioperative period. Fat-free mass (FFM), fat-free mass index (FFMI), skeletal muscle mass (SMM), extracellular water (ECW), total body water (TBW), hydration, and body cell mass (BCM) rise slightly and then fall in the postoperative period in patients with gastrointestinal cancer, and had a sustained increase in non-gastrointestinal patients, respectively (*P*<0.05). Postoperative body composition changes in patients with gastrointestinal cancer are related to preoperative albumin, pre-albumin, hemoglobin, and C-reactive protein (*P*<0.05), whereas postoperative body composition changes in patients with non-gastrointestinal cancer are related to age (*P*<0.05).

**Conclusions:**

Significant changes in body composition both in patients with gastrointestinal cancer and non-gastrointestinal cancer during perioperative period are observed. Changes in body composition for the cancer patients who undergoing surgery are related to age and preoperative serum nutrition index.

## 1 Introduction

Cancer is the second leading cause of death in the world and an important barrier to increasing life expectancy in China, placing a heavy burden on economic and public health systems (1). Over the past 35 years, the incidence and mortality rate of liver and stomach cancers have remained high, while that of lung, breast, colorectal, and prostate cancers has been growing rapidly in China (2).

The utilization of bioelectrical impedance analysis (BIA) for measuring body composition has generated significant interest in using various indicators, including skeletal muscle index (SMI), phase angle (PA), fat mass (FM), fat-free mass (FFM), fat-free mass index (FFMI), cellular water, to predict outcomes in patients with lung cancer (3, 4), breast cancer (5), prostate cancer (6), gastric cancer (7) and colorectal cancer (8). In contrast to traditional BIA methods, bioelectrical impedance vector analysis BIVA can provide more objective information about hy and BCM (9). Such equipment is faster, more portable, and provides more information for diagnosing malnutrition compared to cross-sectional imaging and has been proven good agreement with body composition data provided by the computed tomography (CT) (10) and dual-energy X-ray absorptiometry (DXA) (11) methods. Malnutrition is associated with disease progression and cancer treatments, with negative impacts on quality of life, high grade state of inflammation (12), poor tolerance to antineoplastic treatment and decreased survival, in addition to increasing postoperative complications (13), hospital stay and costs (14). Tumor subsite is one of the major risks of malnutrition, with cancers that affect gastrointestinal function having the highest prevalence (75% for gastroesophageal and 70.6% for pancreatic tumors). And the risk of malnutrition in patients with non-gastrointestinal tumors is significantly reduced (26.6–42.9% for lung tumors and 28.6% for prostate/testicle neoplasms), especially since prevalence is lower in patients with breast cancers (15, 16).

It is important to note that cancer treatments, including surgery, may further change body composition, and increase the risk of malnutrition. Patients with operable colorectal cancer showed a significant decrease in current body weight, FM, and visceral fat score and increased the average percentage of SMI and total water content at 3 months after surgery (17). Fredrix et al. were documented that after surgical removal of the tumor 1 year in non-small cell lung carcinoma patients with FM and FFM increased (18). For patients with gastric cancer, FFM and SMI were significantly decreased at 18 to 24 months after operative treatment (19), moreover, lean body mass after gastrectomy had a greater decrease in elderly (≥80 years old) than in non-elderly patients (<80 years old) (20). The above literature describes body composition changes 3-12 months or even more after malignant tumor surgery, and few literatures reported short-term body composition changes during hospitalization. Previous studies had reported that post-operation 1 week loss of lean body mass was significantly greater than the loss of fat mass in gastric cancer patients (21). Moreover, the changes in water distribution, PA, initial reduced muscle function, and altered biochemical values during the first 9 postoperative days were observed in patients after pancreatic surgery (22). However, few studies have reported a comparative analysis of body composition changes during surgical treatment in patients with gastrointestinal cancer and non-gastrointestinal cancer, which provide useful information for cancer prognosis and more precise nutritional support.

In this study, we performed serial evaluations of the body composition changes during surgical treatment using a bioelectrical impedance vector analyzer and compared the changes and contributing factors in body composition between patients with gastrointestinal cancer and non-gastrointestinal cancer.

## 2 Materials and methods

### 2.1 Study design and patients

Patients inclusion criteria were as follows (1): age 18 years or older, conscious and able to cooperate with relevant inspection; (2) a histologic or clinical diagnosis of lung cancer, breast cancer, prostatic cancer, stomach cancer, and colorectal cancer; (3) complete medical history records are available; (4) patients without severe and vital organ failure (heart, lung, liver, kidney, etc.) or Acquired Immune Deficiency Syndrome AIDS; (5) patients without a cardiac pacemaker or implanted medical device; (6) patients who did not require dialysis or received intravenous fluids within 1 hour before measurement intravenous line; (7) patients without severe pleural effusion and ascites. The prospective observational cohort study according to the above inclusion criteria finally included 92 patients with diagnoses of lung cancer (N=18), breast cancer (N=16), prostatic cancer (N=20), stomach cancer ((N=19), and colorectal cancer (N=19), who were scheduled to receive surgery from September 2022 to December 2022 at Shaoxing People’s Hospital (Shaoxing Hospital Zhejiang University School of Medicine).

General information including age, sex, grip strength, weight, and height was collected. Biochemical profiles and medical information were collected from electronic medical laboratory records. Body composition measurement was performed on preoperative day 1, postoperative day 1, and 1 day before discharge. And nutritional state assessment was performed within 24 hours of admission and before discharge (day -1). All the measurements and information collection were performed by well-trained researchers.

### 2.3 Anthropometry and body composition measurement

Height and body weight was measured using a calibrated stand-up scale and body mass index (BMI) was calculated according to the formula weight (kg)/height (m^2^). Patients were in a standing position with the elbow fully extended and dominant hand-grip strength was measured 3 times by CAMRY EH101 dynamometer, with a maximum squeeze of at least 5s, and with a 30s gap between 3 trials, the maximum value was taken. Regard Jamar dynamometer as the reference device, Camry Digital Handgrip Dynamometer is a valid tool for assessing grip strength in hospitalized adult patients (23).

Body composition analysis was performed using the Bioelectrical Impedance Analysis (BIA, NUTRILAB, AKERN, Italy) which applies alternating sinusoidal electric currents of 400 µA at an operating frequency of 50 kHz. The measurement was performed on preoperative day 1, postoperative day 1, and 1 day before discharge in the morning (8:30-10:00 a.m.).

Patients removed all metal objects and other items that might interfere with the scan and were lying supine on a bed for at least 5 minutes with their legs separated and arms abducted from the body. This method requires only the placement of two single use electrodes on the dorsal surface of the right hand/wrist and the other two on the right foot/ankle attaching leads according to the manufacturer’s instructions. Specific data of sex, age, height, and current weight were added to the machine before starting the impedance. The following parameters were obtained: FM, FFM, PA, FFMI, skeletal muscle mass (SMM), extracellular water (ECW), total body water (TBW), hydration, and BCM.

### 2.4 Nutritional statuses assessment scale

All patients had malnutrition screening by the nutritional risk screening 2002 (NRS2002). NRS2002 with a total score ranging from 0 to 7 points, which has been proven to be a reliable tool for assessing malnutrition risk according to patients’ nutritional status and disease severity. A score of ≥3 points is considered to be at risk of malnutrition (24).

Patient-generated subjective global assessment (PG-SGA) has been shown to be a strong predictor of malnutrition in cancer patients, based on objective indicators such as medical and dietary history (weight change, food intake, two or more weeks of continued gastrointestinal abnormality, and physical function) provided by the patient and combined with clinical examination (body fat loss, muscle mass loss, existence of edema, and hydrops abdominis) to assess the nutritional status of cancer patients. Higher scores (≥9) reflect higher risks of malnutrition (25).

Global Leadership Initiative on Malnutrition (GLIM) criteria (26), which include three phenotypic criteria (weight loss, low BMI, and reduced muscle mass) and two etiologic criteria (reduced food intake and disease burden/inflammation). As one of the GLIM diagnostic criteria for malnutrition, body composition shows its importance in the assessment of nutritional status. Patients are diagnosed with malnutrition at least present one of the phenotypic criteria and one of the etiological criteria. Higher scores (2 points) indicate that the patient has a primary diagnosis of malnutrition.

### 2.5 Biochemical profile and medical information

Biochemical values including serum levels of albumin (Alb), prealbumin (pre-Alb), hemoglobin (Hb), and C-reactive protein (CRP), as well as diagnosis, clinical tumor stage, length of stay, and hospital cost were collected from electronic medical laboratory records on admission.

### 2.6 Statistical analysis

Categorical variables were represented as numbers (percentage). Normally distributed continuous variables were reported as mean ± standard deviation (SD) and non-normally distributed continuous variables were reported as median (interquartile range, IQR). The Student’s *t* test and Wilcoxon test were performed to compare the baseline characteristics between the gastrointestinal cancer group and the non-gastrointestinal cancer group, respectively, for normally and non-normally distributed continuous data. Additionally, the Chi-square test was used for the comparison of categorical variables.

Analysis of covariance (ANCOVA) was performed to detect postoperative differences compared to the preoperative values. Changing Trends of 3 times body composition were analyzed by repeated measures of variance (RMANOVA). The results of ANCOVA and RMANOVA were adjusted for baseline age, sex, BMI, grip, GLIM score, Alb, pre-Alb, Hb, and CRP.

Spearman test was used to explore relationships between body composition changes and baseline variables, and the correlation coefficients (r) were presented. *P* values of less than 0.05 were considered statistically significant. BIVA 2002 software was used for the construction of the vectorial plot (9). All results were analyzed using the Statistical Analysis Software (SAS) 9.4 (SAS Institute Inc., Cary, North Carolina, U.S.).

### 2.7 Ethical approval

All patients volunteered to participate in this study and received oral and written information about the project, before asking for their written informed consent. This study did not interfere with the clinical practice in the hospital and was approved by Shaoxing People’s Hospital (Shaoxing Hospital Zhejiang University School of Medicine).

## 3 Results

### 3.1 Baseline characteristics of patients

As shown in **Table 1**, the study included 92 patients (53 males and 39 females) with an average age of 66.76 years old, an average BMI of 23.31 kg/m^2,^ and an average grip of 27.72 kg. The gastrointestinal cancer group included 26 males and 12 females with an average age of 69.24 years old, an average BMI of 22.98 kg/m^2,^ and an average grip of 28.58 kg. For the non-gastrointestinal cancer group, 27 males and 27 females were included and with an average age of 65.02 years old, an average BMI of 23.55 kg/m^2,^and an average grip of 27.11 kg. No significant difference in age, sex, BMI, and grip between the gastrointestinal cancer group and non-gastrointestinal cancer group (*P*>0.05). A majority of patients (68.48%) were diagnosed with an early (1 or 2) cancer stage. A total of 4 patients with TNM (tumor, node, and metastasis) stage IV were included, all of them from the gastrointestinal cancer group. From a preoperative assessment, 17 patients (18.68%) had a malnutrition diagnosis (GLIM=2 points). The blood biochemical index including Alb, pre-Alb, and Hb in patients with gastrointestinal cancer was significantly lower than that of patients with non-gastrointestinal cancer (*P*<0.05), whereas the CRP was significantly higher in the gastrointestinal cancer group (*P*=0.0002). Additionally, longer stays (median=16, IQR=14-19 vs. median=9, IQR=7-12) and more cost (median=44927.8, IQR=37825.6-51565.80 vs. median=22366.94, IQR=19661.52-29072.42) for the patients with gastrointestinal cancer compared to the non-gastrointestinal cancer group (*P*<0.0001).

**Table 1:** Basic characteristics of patients.

Baseline demographics	Total	Gastrointestinal Cancer group	Non-gastrointestinal Cancer group	*P* value
**Patients (N)**	92	38	54	
**Age (year) (Mean ± SD)**	66.76±11.21	69.24±9.84	65.02±11.86	0.1465
**Sex, N (%)**				0.0783
Male	53 (57.61)	26 (68.42)	27 (50.00)	
Female	39 (42.39)	12 (31.58)	27 (50.00)	
**BMI (kg/m^2^) (Mean ± SD)**	23.31±3.43	22.98±3.80	23.55±3.17	0.1948
**Grip (Kg) (Mean ± SD)**	27.72±9.17	28.58±10.13	27.11±8.48	0.5388
**TNM stage, N (%)**				**<.0001**
I	42 (45.65)	11 (28.95)	31 (57.41)	
II	21 (22.83)	5 (13.16)	16 (29.63)	
III	25 (27.17)	18 (47.37)	7 (12.96)	
IV	4 (4.35)	4 (10.53)	0 (0)	
**GLIM, N (%)**				**0.0075**
1 point	74 (81.32)	26 (68.42)	48 (90.57)	
2 points	17 (18.68)	12 (31.58)	5 (9.43)	
**Biochemical values (Mean ± SD)**				
Albumin (g/l)	40.52±5.78	36.79±4.99	43.23±4.74	**<.0001**
Prealbumin (mg/l)	231.44±68.27	195.73±66.38	256.17±58.34	**<.0001**
C-reactive protein (mg/l)	9.74±29.69	15.75±27.96	5.47±30.4	**0.0002**
Hemoglobin (g/l)	131.87±23.26	126.7±21.90	135.36±23.72	**0.0117**
**Length of hospital stay (day),** **Median (IQR)**	12 (8 to 16)	16 (14 to 19)	9 (7 to 12)	**<.0001**
**Hospitalization cost (yuan),** **Median (IQR)**	31123.90 (22037.14 to 42922.85)	44927.8 (37825.6 to 51565.80)	22366.94 (19661.52 to 29072.42)	**<.0001**

BMI, body mass index; TNM, tumor, node, and metastasis; GLIM, the Global Leadership Initiative on Malnutrition. Bold values indicate that the difference is statistically significant, significance level P<0.05.

### 3.2 Changes in body composition, NRS2002, and PA-SGA during the perioperative period

The absolute values for body composition and nutrition statement during the perioperative period are shown in **Table 2**. Overall, after adjusted age, sex, grip, BMI, TNM stages, Alb, pre-Alb, CRP, and Hb by ANCOVA, significant perioperative changes were found in all patients. For the gastrointestinal cancer group, FFM, FFMI, and SMM increased modestly after surgery (FFM+1.39, SD=2.60; FFMI+0.47, SD=0.83; SMM+1.33, SD=2.37) (*P*<0.05) but declined significantly on 1 day before discharge (FFM-0.27, SD=2.32; FFMI-0.05, SD=0.81; SMM-0.12, SD=2.24) (*P*<0.05). FM decreased on postoperative day 1 (-1.39, SD=2.60) (*P*=0.0011) and increased on 1 day before discharge (+0.27, SD=2.32) (*P*<0.0001). PA value reduced significantly on 1 day after surgery (-0.15, SD=0.75) (*P*=0.0029) and remained low on 1 day before discharge (-0.22, SD=0.82) (*P*=0.0007). Body water compartment changes were observed. In particular, ECW and hydration increased significantly on postoperative day 1 (ECW+0.97, SD=2.35; hydration+0.56, SD=2.05) (*P*<0.05) and, despite a small reduction, remained higher than the preoperative value before discharge (ECW+0.41, SD=2.17; hydration+0.30, SD=2.11) (*P*<0.05). Whereas TBW and BCM increased slightly after surgery (TBW+1.32, SD=2.41; BCM+0.29, SD=2.21) (*P*<0.05) but fell significantly on 1 day before discharge (TBW-0.14, SD=2.25; BCM-0.77, SD=2.23) (*P*<0.05). For the non-gastrointestinal cancer group, FFM, FM, TBW, ECW, and BCM increased on postoperative day 1, and 1 day before discharge (postoperative day 1:FFM+1.37, SD=3.03; FM-1.37, SD=3.03; TBW+1.06, SD=2.41; ECW+0.59, SD=1.65; BCM+0.58, SD=2.81; 1 day before discharge: FFM+1.36, SD=3.42; FM-1.38, SD=3.42; TBW+1.61, SD=4.79; ECW+1.07, SD=2.09; BCM+0.22, SD=3.67) (*P*<0.05). FFMI and SMM increased significantly on postoperative day 1 (FFMI+0.41, SD=0.87; SMM+1.07, SD=2.38) (*P*<0.05) and recovered before discharge (FFMI+0.63, SD=1.92; SMM+1.70, SD=5.37) (*P*>0.05).

**Table 2:** Body composition, NRS2002 and PA-SGA values during the perioperative period.

	preoperative day 1	postoperative day 1	1 day before discharged	*P* ^a^ value	*P* ^b^ value	*P* ^c^ value
Gastrointestinal Cancer						
BIVA parameter						
FFM (kg)	49.94 ± 9.37	51.33±9.49	49.67±9.02	**<.0001**	**<.0001**	**0.0174**
FM (kg)	12.80±7.40	11.41±7.48	13.07±6.88	**0.0011**	**<.0001**	**0.0164**
PA (°)	5.68±0.85	5.53±0.92	5.46±1.12	**0.0029**	**0.0007**	0.9366
FFMI (kg/m^2^)	9.02±1.69	9.48±1.76	8.97±1.51	**0.0002**	**0.0003**	0.0515
SMM (kg)	24.86±6.27	26.19±6.60	24.74±5.86	**<.0001**	**<.0001**	**0.0465**
TBW (L)	36.91±7.09	38.22±7.37	36.77±6.71	**<.0001**	**<.0001**	0.0559
ECW (L)	17.52±3.51	18.48±3.92	17.92±3.55	**<.0001**	**<.0001**	0.1012
Hydration	73.75±2.88	74.31±3.39	74.06±3.45	**<.0001**	**<.0001**	0.2260
BCM	25.97±6.15	26.26±6.41	25.19±6.73	**<.0001**	**<.0001**	0.2707
Nutrition statement						
NRS2002	2.18±1.23	–	3.89±1.48	–	**<.0001**	–
PG-SGA	3.92±3.47	–	9.56±2.43	–	**<.0001**	–
Non-gastrointestinal cancer						
BIVA parameter						
FFM (kg)	49.45±8.84	50.82±9.40	50.81±9.74	**<.0001**	**<.0001**	**0.0174**
FM (kg)	13.42±5.54	12.05±5.70	12.04±6.19	**<.0001**	**0.0002**	**0.0164**
PA (°)	5.91±0.77	5.85±0.65	5.72±0.89	0.2442	0.4769	0.9366
FFMI (kg/m^2^)	8.80±1.60	9.21±1.78	9.43±2.30	**<.0001**	0.9162	0.0515
SMM (kg)	23.78±5.87	24.85±6.33	25.49±7.70	**<.0001**	0.1053	**0.0465**
TBW (L)	36.28±6.56	37.34±7.08	37.89±7.86	**<.0001**	**0.0031**	0.0559
ECW (L)	16.73±2.73	17.31±3.01	17.80±3.04	**<.0001**	**0.0095**	0.1012
Hydration	73.16±0.92	73.34±0.65	73.77±1.90	0.0961	0.5611	0.2260
BCM	26.38±5.92	26.96±5.90	26.60±6.57	**<.0001**	**0.0007**	0.2707
Nutrition statement						
NRS2002	1.46±0.50	–	1.45±0.50	–	0.8997	–
PG-SGA	1.66±0.61	–	4.79±2.40	–	**0.0003**	–

Values expressed as Mean±SD. BIVA, bioelectric impedance vector analysis; FFM, fat free mass; FFMI, fat free mass index; FM, fat mass; PA, phase angle; SMM, skeletal muscle mass; TBW, total body water; ECW, extracellular water; BCM, body cell mass; NRS2002, the nutritional risk screening 2002; PG-SGA, the patient generated subjective global assessment.

*P* ^a^: ANCOVA for postoperative day vs. 1 preoperative day 1;

*P* ^b^: ANCOVA for 1 day before discharged vs. 1 preoperative day 1;

*P* ^c^: RMANOVA for the changes of body composition during perioperative period between gastrointestinal cancer group and non-gastrointestinal cancer group.

The results of ANCOVA and RMANOVA were adjusted for baseline age, sex, body mass index, grip, GLIM, albumin, prealbumin, hemoglobin and C-reactive protein. Bold values indicate that the difference is statistically significant, significance level P<0.05.

With regard to the nutrition statement, all of the patients’ PG-SGA scores increased significantly on 1 day before discharge compared to preoperative (gastrointestinal cancer group+5.64, SD=2.14; non-gastrointestinal cancer group+3.13, SD=2.03) (*P*<0.001), and the NRS2002 score was elevated only observed in patients with gastrointestinal cancer (+1.71, SD=1.20) (*P*<0.0001).

**Figure 1:**
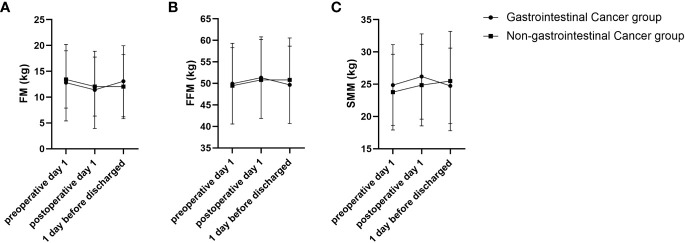
FM, FFM and SMM trajectories of changes during perioperative period of gastrointestinal cancer group and non-gastrointestinal cancer group. **(A)** FM trajectory of changes during perioperative of gastrointestinal cancer group and non-gastrointestinal cancer group; **(B)** FFM trajectory of changes during perioperative period of gastrointestinal cancer group and non-gastrointestinal cancer group; **(C)** SMM trajectory of changes during perioperative period of gastrointestinal cancer group and non-gastrointestinal cancer group.

**Figure 2:**
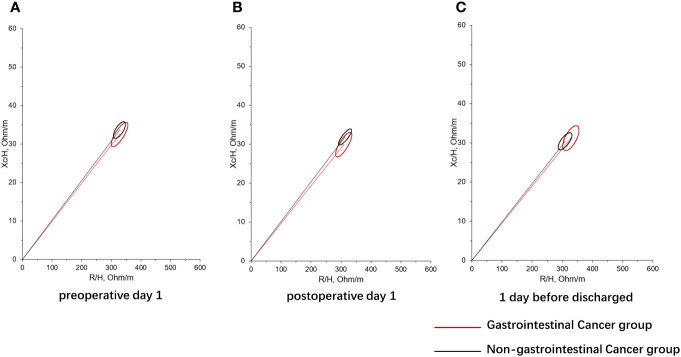
RXc mean graph with the 95% confidence ellipses during perioperative period of gastrointestinal cancer group and non-gastrointestinal cancer group. **(A)** RXc mean graph with the 95% confidence ellipses on preoperative day 1 of gastrointestinal cancer group and non-gastrointestinal cancer group; **(B)** RXc mean graph with the 95% confidence ellipses on postoperative day 1 of gastrointestinal cancer group and non-gastrointestinal cancer group; **(C)** RXc mean graph with the 95% confidence ellipses on 1 day before discharge of gastrointestinal cancer group and non-gastrointestinal cancer group. R is the resistance, Xc is the reactance, and H is the height.

In addition, RMANOVA showed that the changes in FFM, FM, and SMM during the perioperative period between the gastrointestinal cancer group and non-gastrointestinal cancer group were significant differences (*P*<0.05). As shown in **Figure 1**, FFM, FM, and SMM increased slightly in the gastrointestinal cancer group on 1 day after surgery and decreased below the preoperative level before discharge, while SMM kept increasing in the non-gastrointestinal cancer group. Changes in body composition lead to alterations in electrical resistance (R) and reactance (Xc) within the body, which ultimately impact PA, considering that it is the angular transformation of the ratio between Xc and R (27). RXc mean graph with the 95% confidence ellipses during the perioperative period of the gastrointestinal cancer group and non-gastrointestinal cancer group are shown in **Figure 2**. With respect to the non-gastrointestinal cancer group vector, a shorter impedance vector was demonstrated in gastrointestinal cancer group patients both on preoperative day 1 and postoperative day 1, and a longer impedance vector was demonstrated on 1 day before discharge. The 95% confidence ellipses of the two groups were overlapping, which means no significant vector displacement.

### 3.3 Correlations between changes in body composition and baseline characteristics of patients

The correlation coefficient between changes in body composition and baseline characteristics of patients is shown in **Table 3**. For the gastrointestinal cancer group, on postoperative day 1, the changes in PA and BCM were positively correlated with preoperative Alb and pre-Alb level (*P*<0.05), and the changes in BCM were positively correlated with preoperative hemoglobin level(*P*<0.05). A significant negative correlation was found between the changes in hydration and preoperative pre-Alb level (*P*<0.05). No association was observed between CRP and changes in body composition on postoperative day 1 (*P*>0.05). On 1 day before discharge, the changes in PA and BCM were positively correlated with preoperative Alb and pre-Alb levels (*P*<0.05), and a significant positive correlation between BCM and preoperative Hb level was found(*P*<0.05), whereas the changes in ECW was negatively correlated with preoperative Alb and pre-Alb level (*P*<0.05). Preoperative CRP level was positively correlated with changes in FM, FFMI, SMM, TBW, ECW, and hydration (*P*<0.05), and negatively correlated with changes in FM, PA, and BCM (*P*<0.05). No association was observed between age, grip, BMI, TNM stage, and changes in body composition during the period of postoperative to pre-discharge (*P*>0.05).

**Table 3:** Correlations between changes in body composition and baseline characteristics of patients.

	Age	Grip	BMI	Alb	pre-Alb	CRP	Hemoglobin	TNM stage
Gastrointestinal cancer								
Changes are calculated as “measurement on postoperative day 1 - measurement on preoperative day 1”.								
Changes in FFM (kg)	0.05905	0.04443	-0.07004	0.15914	0.03038	0.06924	0.17686	-0.17434
Changes in FM (kg)	-0.05905	-0.04443	0.07004	-0.15914	-0.03038	-0.06924	-0.17686	0.17434
Changes in PA (°)	0.03328	0.19038	-0.13892	0.36270*	0.43468*	-0.12316	0.25422	0.17610
Changes in FFMI (kg/m^2^)	0.20171	-0.12598	-0.04962	0.00380	-0.11044	0.06955	-0.03004	-0.18746
Changes in SMM (kg)	0.18475	-0.11297	-0.01412	0.02823	-0.07919	0.05432	-0.00308	-0.17227
Changes in TBW (L)	0.18091	-0.08670	-0.02354	0.03897	-0.07043	0.03819	0.01732	-0.18456
Changes in ECW (L)	0.14972	-0.17990	-0.00471	-0.21783	-0.26275	0.05765	-0.19553	-0.22624
Changes in Hydration	0.13623	-0.24024	0.02053	-0.24356	-0.35362*	0.02118	-0.25857	-0.19933
Changes in BCM	0.00428	0.27147	-0.18489	0.49712*	0.46701*	-0.20776	0.34476*	-0.03473
Changes are calculated as “measurement on 1 day before discharged - measurement on preoperative day 1”.								
Changes in FFM (kg)	0.04931	-0.09962	0.00996	-0.14524	-0.24271	0.33264*	0.13205	-0.06814
Changes in FM (kg)	-0.04931	0.09962	-0.00996	0.14524	0.24271	-0.33264*	-0.13205	0.06814
Changes in PA (°)	-0.23118	0.27990	0.13189	0.32169*	0.44811*	-0.59432*	0.27745	0.01531
Changes in FFMI (kg/m^2^)	0.13310	-0.16465	0.03464	-0.12014	-0.23624	0.40625*	0.18463	0.00077
Changes in SMM (kg)	0.12229	-0.18369	0.00985	-0.13563	-0.26859	0.40368*	0.15792	-0.01846
Changes in TBW (L)	0.11468	-0.17156	-0.00460	-0.13150	-0.26877	0.41181*	0.15533	-0.02299
Changes in ECW (L)	0.27862	-0.28514	-0.10567	-0.32266*	-0.44144*	0.64721*	-0.17559	-0.07199
Changes in Hydration	0.17930	-0.19369	-0.28010	-0.14339	-0.28688	0.40898*	-0.12813	-0.12741
Changes in BCM	-0.24307	0.28338	0.07356	0.33019*	0.39204*	-0.46555*	0.34393*	-0.12375
Non-gastrointestinal cancer								
Changes are calculated as “measurement on postoperative day 1 - measurement on preoperative day 1”.								
Changes in FFM (kg)	-0.14618	0.03440	0.10206	0.04815	0.19374	0.00141	0.03483	0.04404
Changes in FM (kg)	0.14618	-0.03440	-0.10206	-0.04815	-0.19374	-0.00141	-0.03483	-0.04404
Changes in PA (°)	0.28773*	-0.09232	0.06146	-0.05289	-0.05248	0.09274	0.04754	-0.07639
Changes in FFMI (kg/m^2^)	-0.18468	0.01453	0.11472	0.05455	0.18475	-0.02863	-0.01844	-0.00127
Changes in SMM (kg)	-0.17193	0.03586	0.11369	0.06130	0.18479	-0.00980	0.01010	0.01342
Changes in TBW (L)	-0.16821	0.04986	0.11146	0.04985	0.18525	0.00653	0.02099	0.01689
Changes in ECW (L)	-0.36911*	0.04186	0.03578	0.02525	0.16034	-0.00970	-0.04845	-0.02942
Changes in Hydration	-0.33026*	-0.09618	0.03747	0.03890	0.06627	-0.06383	-0.12733	-0.02815
Changes in BCM	0.07399	-0.00664	0.15451	0.03138	0.13022	0.06900	0.05766	-0.01991
Changes are calculated as “measurement on 1 day before discharged - measurement on preoperative day 1”.								
Changes in FFM (kg)	-0.13099	0.15318	-0.01615	-0.01583	0.14786	-0.01346	-0.02531	0.08910
Changes in FM (kg)	0.13273	-0.13223	0.03177	0.02568	-0.13013	0.02247	0.02291	-0.07797
Changes in PA (°)	0.20948	-0.01805	-0.01474	-0.09527	-0.01009	-0.00934	0.01508	0.16397
Changes in FFMI (kg/m^2^)	-0.15552	0.08645	-0.12107	-0.06395	0.05294	0.01165	-0.16758	0.01142
Changes in SMM (kg)	-0.15404	0.12532	-0.10539	-0.06647	0.07057	0.03959	-0.11158	0.04275
Changes in TBW (L)	-0.15087	0.13073	-0.09360	-0.06402	0.07634	0.03791	-0.10839	0.04163
Changes in ECW (L)	-0.22461	0.00574	-0.05673	-0.02606	0.02044	0.10237	-0.12981	-0.12020
Changes in Hydration	-0.26398*	-0.10102	-0.09558	0.00518	0.00609	0.06882	-0.21382	-0.14554
Changes in BCM	0.02861	0.05901	0.00608	-0.05091	0.11440	-0.04125	-0.02498	0.08054

Values expressed as correlation coefficients (r). TNM, tumor, node, and metastasis; FFM, fat free mass; FFMI, fat free mass index; FM, fat mass; PA, phase angle; SMM, skeletal muscle mass; TBW, total body water; ECW, extracellular water; BCM, body cell mass; bold values indicate that the difference is statistically significant, significance level P<0.05.

For the non-gastrointestinal cancer group, on postoperative day 1, we found changes in ECW and hydration had a slight, negative, and significant correlation with age (*P*<0.05), and a significant positive correlation was found between the changes in PA and age (*P*<0.05). On 1 day before discharge, the changes in hydration were negatively associated with age(*P*<0.05). No association was observed between grip, BMI, Alb, pre-Alb, CRP, Hb, TNM stage, and changes in body composition during the period of postoperative to pre-discharge (*P*>0.05).

## 4 Discussion

In the present study, we prospectively analyzed the changes in body composition during the perioperative period with operable patients who were diagnosed with lung, breast, prostate, gastric and colorectal cancers, and divided them into a gastrointestinal cancer group (gastric cancer and colorectal cancer) and non-gastrointestinal cancer group (lung cancer, breast cancer and prostate cancer) to compare differences. The results showed all patients in the study had changes in body composition throughout the hospitalization, the trajectories of FM, FFM, and SMM were significantly different between groups. And the changes in body composition of the gastrointestinal cancer group and non-gastrointestinal cancer group were related to preoperative serum markers of nutrition and age, respectively. To the best of our knowledge, our study is the first to use BIVA to find changes in body composition in patients operated for gastrointestinal and non-gastrointestinal cancer among the Chinese.

Our results indicate that the trajectories of FM, FFM, and SMM were minor increased after surgery and then decreased to below the preoperative level, while the non-gastrointestinal cancer group consistently increased before hospital discharge. This means that the nutritional status of patients with non-gastrointestinal cancer is less affected by surgery, while patients with gastrointestinal cancers have worse nutritional status after surgery. Moreover, patients with longer postoperative stays in bed and lack of exercise had more muscle atrophy and lost significant FFM at the time of discharge for gastrointestinal cancer patients. In addition, the short-term decrease in FM may be related to the accelerated rate of protein catabolism and metabolism caused by the stressful trauma of surgery, the postoperative fasting of patients, the slow recovery of digestive tract function, and the relative lack of nutrition supplemented by food intake (28). In contrast, patients with non-gastrointestinal cancer are less affected by postoperative food intake and activity limited.

Skeletal muscle contributes to systemic effects by secreting cytokines and other myokines (including IL-6, IL-8, IL-15, and leukemia inhibitory factors) through local autocrine, paracrine, and endocrine actions (29). Therefore, lower levels of muscle associated with longer length of stay, higher risk of postsurgical complications and mortality (30), as well as lead to local and systemic inflammation (31), which may enhance catabolism, lead to ongoing muscle loss in cancer patients and associations with cancer survival (32, 33). Supplementation with whey protein, branched-chain amino acid, and vitamin D is not only beneficial for maintaining muscle mass (34), in conjunction with age-appropriate exercise, but also boosts FFM and strength that contribute to well-being in patients (35).

Researchers have considered that cellular hydration plays a protective role against weakness, frailty status and functional decline (36). We observed that the hydration status of the gastrointestinal cancer group temporarily increases after surgery, then declines and remains below preoperative levels. The non-gastrointestinal group had increases in TBW, ECW, and hydration, and this state occurred after surgery and persisted for the remainder of the study period. There is evidence that the cellular hydration state is an important factor controlling cellular protein turnover, protein synthesis and protein degradation are affected in opposite directions by cell swelling and shrinking (37). An increase in cellular hydration (swelling) acts as an anabolic proliferative signal, and loss of plasma fluid and small proteins leads to a decreased plasma capillary oncotic pressure, with ongoing interstitial leakage of fluid and electrolytes resulting in localized edema, which is increased as inflammation impedes the reabsorption and return of fluid to the circulation *via* the lymphatics (38). Our results showed that the higher the preoperative CRP, the less the decrease in FFM, FFMI, and SMM and the more the increase in ECW before discharge, suggesting that the preoperative inflammatory status of patients with gastrointestinal cancer may influence the distribution of hydration after operation. FFM contains virtually all the water and conducting electrolytes in the body, and its hydration is constant (39). Thus, high preoperative CRP levels and a smaller postoperative decrease in FFM may be associated with an increase in postoperative ECW. The shift in water distribution reflects the depletion of BCM (40), which indicates the impairment of organ function in patients with malignant tumors and affects patient prognosis (41). Moreover, we observed an increase in changes in hydration declines with age in the non-gastrointestinal tumor group. Risks of dehydration increase with advancing age (42), mechanistically, dehydration yields stable metabolism remodeling, an elevation of markers of inflammation and coagulation, and renal glomerular injury (43). Improving hydration throughout life may greatly decrease the prevalence of degenerative diseases relate to age.

PA is a sign of cell membrane health and integrity, hydration, and nutritional status. Previous studies have demonstrated that PA is a predictor of mortality or postoperative complications in different clinical settings (27). Although the vector did not shift significantly in either group of subjects, we observed a shorter impedance vector in the gastrointestinal cancer group with respect to the non-gastrointestinal cancer group on preoperative day 1 and postoperative day 1 (**Figure 2**). The present findings indicate a significant decrease of PA after surgery both in two groups, suggesting decreased cellular integrity and poorer nutritional status in them. Consistent with previous studies (44, 45), the changes of PA with serum Alb and pre-Alb were observed a remarkable positive correction on postoperative day 1 and before discharge in patients with gastrointestinal cancer. In addition, BCM showed similar postoperative changes to PA. It is suggested that postoperative PA and BCM loss decreases with the increase of preoperative serum nutrient parameters in patients with gastrointestinal cancer. Previously, Barrea et al. (46) documented that PA represents a valid predictor of CRP levels in both sexes regardless of body weight, and is possible to predict nutrition-related inflammation. On 1 day before discharge, we also observed a negative association between the changes of PA and CRP in the gastroenteric cancer group. High CRP before surgery may result in a significant decrease in PA. Therefore, preoperative nutritional supplementation should be encouraged in routine practice in patients undergoing operations for gastrointestinal cancer, which is helpful to suppress perioperative inflammation, improve the postoperative nutritional status, and reduce postoperative infection complications (47, 48). Additionally, body composition can inform the formulation of preoperative nutritional therapy for patients. Although no relationship was found between changes in body composition and baseline characteristics of patients with non-gastroenteric cancer in the present study, preoperative nutrition is also important for them (49).

PG-SGA is a tool to effectively assess the nutritional status of oncology patients (25). Compared to preoperative, the patients in this study all had higher PG-SGA scores and showed significant changes in body composition after surgical treatment. It is evident that body composition is an important component of the comprehensive nutritional evaluation of oncology patients, and this result is consistent with other studies (50, 51). Body composition measures can be a more effective predictor of the malnutrition than BMI or body weight and should be considered as part of preoperative risk management and when designing nutritional interventions for undergoing surgery cancer patients. The above findings indicated that the postoperative body composition of patients with malignant tumors is not only related to tumor type, but also significantly correlated with preoperative nutritional status and age.

Although the BIVA method is not considered the “gold standard” for assessing body composition, it has been shown to provide information on hydration and BCM, which allows for the evaluation of patients in whom we are unable to accurately extrapolated body composition due to altered hydration (52). This body composition measurement is useful in guiding the development of nutritional treatment.

This is the first study to use BIVA to identify early postoperative changes of body composition in Chinese patients undergoing surgery for gastrointestinal and non-gastrointestinal cancers, as a way to provide a foundation for personalized nutritional support during hospitalization. Understanding changes in body composition benefits personalized nutritional support and fluid rehydration programs for perioperative patients. For patients undergoing surgery for gastrointestinal, there is a significant loss and a slow recovery in FFM. Hence, it is important to focus on protein supplementation to prevent hypoproteinemia and excessive consumption of FFM during the perioperative period. For patients undergoing surgery for non-gastrointestinal cancer, it is crucial to prioritize correct fluid rehydration during the perioperative period. This study has several limitations. Although we included multiple cancer patients, the sample size for each cancer was small. A larger sample will be needed to collect more accurate data and make more precise conclusions. We only excluded patients who were diagnosed with abdominal fluid and did not define abnormal hydration status by changes in skinfold thickness, heart rate, blood pressure, and hematological and urine parameters, which can be due to the change in TBW. In addition, there was no data on body composition in patients with non-malignant tumors, so we cannot compare the difference in body composition between cancer patients and patients with non-malignant tumors. It would be helpful for subsequent comparisons if data of body composition closer to normal were available.

## 5 Conclusions

We observed significant changes in the early postoperative body composition both in patients with gastrointestinal cancer and non-gastrointestinal cancer after radical resection tumor surgery. Postoperative body composition changes in patients with gastrointestinal cancer are related to preoperative Alb, pre-Alb, CRP, and Hb, whereas postoperative body composition changes in patients with non-gastrointestinal cancer are related to age.

## Data availability statement

The raw data supporting the conclusions of this article will be made available by the authors, without undue reservation.

## Ethics statement

The studies involving humans were approved by Shaoxing People’s Hospital (Shaoxing Hospital Zhejiang University School of Medicine). The studies were conducted in accordance with the local legislation and institutional requirements. The participants provided their written informed consent to participate in this study.

## Author contributions

BC: data collection, formal analysis and writing original draft. LL: data collection, conceptualization, validation, and writing review and editing. CZ: conceptualization, validation, and writing review and editing. LM: supervision and writing review and editing. QS: data collection, writing review and editing. YF: data collection. MW: conceptualization, supervision, methodology and writing review and editing. SC: project administration, conceptualization, methodology, supervision and data visualization. All authors contributed to the article and approved the submitted version.
