# Corrigendum: Influence of body composition assessment with bioelectrical impedance vector analysis in cancer patients undergoing surgery

**DOI:** 10.3389/fonc.2024.1378338

**Published:** 2024-03-14

**Authors:** Bin Cai, Lan Luo, Chenping Zhu, Liping Meng, Qing Shen, Yafei Fu, Mingjie Wang, Sue Chen

**Affiliations:** ^1^ Department of Quality Management, Sir Run Run Shaw Hospital, Zhejiang University School of Medicine, Hangzhou, Zhejiang, China; ^2^ Department of Clinical Nutrition, Shaoxing People’s Hospital, Shaoxing, Zhejiang, China; ^3^ School of Medicine, Shaoxing University, Shaoxing, Zhejiang, China

**Keywords:** body composition, bioelectrical impedance vector analysis, gastrointestinal cancer, nutritional status, malnutrition

In the published article, there was an error. The model of the instrument of body composition analysis and alternating sinusoidal electric was wrong.

A correction has been made to **2. Materials and methods**, *2.3 Anthropometry and body composition measurement*, Paragraph 2. This sentence previously stated:

“Body composition analysis was performed using the bioelectrical impedance vector analysis method with BIA 101 BIVA^®^ PRO instrument (Akern/RJL) which applies alternating sinusoidal electric currents of 250 µA at an operating frequency of 50 kHz.”

The corrected sentence appears below:

“Body composition analysis was performed using the Bioelectrical Impedance Analysis (BIA, NUTRILAB, AKERN, Italy) which applies alternating sinusoidal electric currents of 400 µA at an operating frequency of 50 kHz.”

The authors apologize for this error and state that this does not change the scientific conclusions of the article in any way. The original article has been updated.

